# Attitudes and practices in the management of attention deficit hyperactivity disorder among Brazilian pediatric neurologists who responded to a national survey: a cross-sectional study

**DOI:** 10.1590/1516-3180.2021.0966.R1.20092022

**Published:** 2022-12-19

**Authors:** Marina Estima Neiva Nunes, Patricia Aparecida Zuanetti, Ana Paula Andrade Hamad

**Affiliations:** I MD. Child Neurologist, Department of Neurosciences and Behavioral Sciences, Faculdade de Medicina de Ribeirão Preto (FMRP), Universidade de São Paulo (USP), Ribeirão Preto (SP), Brazil.; II PhD. Speech and Hearing Therapist, Department Health Sciences, Hospital das Clínicas da Faculdade de Medicina de Ribeirão Preto (HCFMRP), Universidade de São Paulo (USP), Ribeirão Preto (SP), Brazil.; III MD, PhD. Child Neurologist, Department of Neurosciences and Behavioral Sciences, Faculdade de Medicina de Ribeirão Preto (FMRP), Universidade de São Paulo (USP), Ribeirão Preto (SP), Brazil.

**Keywords:** Attention deficit disorder with hyperactivity, Child behavior disorders, Neurodevelopmental disorders, ADHD treatment, Psychostimulants, Attention deficit disorder, Hyperkinetic syndrome

## Abstract

**BACKGROUND:**

Attention deficit hyperactivity disorder (ADHD) has a prevalence of 5.3% among children and adolescents. It is characterized by attention deficit, hyperactivity, and impulsivity.

**OBJECTIVE:**

We aimed to conduct a survey involving pediatric neurologists in the management of ADHD and compare the results with the current literature and guidelines.

**DESIGN AND SETTING:**

Descriptive analytical study of a virtual environment, was used Test of equality of proportions for comparison between two groups of pediatric neurologists (working as specialists for > 6 versus ≤ 6 years), with a significance level of P = 0.05.

**METHODS:**

This cross-sectional study used a virtual questionnaire covering the steps in the diagnosis and treatment of children with ADHD. The inclusion criteria were professionals who had completed their residency/specialization in pediatric neurology and clinical neurologists working in pediatric neurology.

**RESULTS:**

Among the 548 electronic invitations sent, 128 were considered valid. For all participants, the diagnosis was clinically based on the disease classification manuals. Combination treatment promotes improvement of symptoms (96.9%). Among psychostimulants, short-acting methylphenidate was the most commonly prescribed medication (85.2%). Headache was the most common side effect (77.3%). Altogether, 73.4% of the participants requested laboratory tests, 71.1% requested an electrocardiogram, and 42.2% requested an electroencephalogram. Pediatric neurologists working as specialists for ≤ 6 years had more frequent referrals to psycho-pedagogists for diagnosis (P = 0.03).

**CONCLUSIONS:**

The participants complied with clinical guidelines, emphasizing the relevance of diagnostic manuals and treatment guidelines for an eminently clinical situation and enabling uniformity in quality treatment.

## INTRODUCTION

Attention deficit hyperactivity disorder (ADHD) is a neurodevelopmental disorder with a global prevalence of 5.3% among children and adolescents (95% confidence interval [CI], 2.6–4.5).^1,2^

Symptoms of ADHD include inattention and excessive hyperactivity/impulsivity for the age or level of development; with impaired personal, social, and academic functioning. Due to the absence of biomarkers, diagnostic criteria focus on behavioral symptoms.^3^ According to the Centers for Disease Control and Prevention, ADHD is the most prevalent neuropsychiatric disorder in childhood and adolescence.^4^ It is the most common behavioral disorder encountered by pediatric neurologists in clinical practice.^1^

## OBJECTIVE

We aimed to conduct a survey involving pediatric neurologists to obtain information on diagnostic and therapeutic management of ADHD and to compare the results with the current literature and guidelines.

## METHODS

After the study was approved by the Ethics Committee on February 20, 2018 (Research Ethics Committee approval number: 2,501,524), questionnaires were sent to pediatric neurologists. We performed a cross-sectional study using a questionnaire based on literature and consisting of 28 multiple-choice questions covering the following topics related to ADHD: diagnosis, pharmacological treatments, and non-pharmacological treatments for children with ADHD in the last 12 months of outpatient clinical care. A tool was used to guide the participants while filling out the questionnaire, making it impossible to move to the next page without properly filling out the current page, thereby preventing incomplete questionnaires.

The questionnaire was adapted to the Google Docs virtual platform and sent twice via e-mail and WhatsApp message to pediatric neurologists registered with the Brazilian Society of Pediatric Neurology. The time required to complete the questionnaire was approximately 10 minutes. No incentive was offered for participation and the questionnaire was only available in Brazilian Portuguese, the official language of Brazil.

The study included professionals who had completed their residency/specialization in pediatric neurology and clinical neurologists working in pediatric neurology. Specialists with laboratory conflicts of interest, professionals working as specialists for < 2 years, and professionals not working in Brazil were excluded.

The results obtained from the questionnaire were analyzed using descriptive statistics. In addition, the test of equality of proportions was used to compare two groups of pediatric neurologists (dichotomized according to the median time they worked as specialists), with a significance level of P = 0.05. In this test, the following variables were analyzed: request for evaluation and/or therapy with a multidisciplinary team (psychologists, psycho-pedagogists, speech therapists, occupational therapists); medical treatment in patients under 6 years of age (preschool age group), complementary tests before the beginning of treatment, option of performing continuous or intermittent treatment, and perception of symptomatic improvement in the face of the proposed treatment (medication with or without therapy with a multidisciplinary team).

## RESULTS

### Characterization of the participants

The data collection phase included 548 members of the Brazilian Society of Pediatric Neurology. Altogether, 788 invitations were sent including 548 by e-mail and 240 via messaging apps to reach 60 members with inactive and/or non-existent e-mails.

Altogether, 150 questionnaires were answered (27.4% of the 548 neuropediatricians). Twenty-two participants (14.7%) were excluded (20 participants worked as specialists for < 2 years, one participant incorrectly filled out the identification details and could not be confirmed by the Federal Council of Medicine, and one was a foreigner). Thus, 128 questionnaires were included in the study.

The participants’ ages ranged from 29 to 74 years, with a mean age of 40.8 years (standard deviation [SD]: 8.9). The duration of working as a specialist ranged from 2 to 46 years, with a mean duration of 10.6 years (SD: 10.1) and a median duration of 6 years. Altogether, 106 participants (82.8%) reported part-time employment in the public health system (mainly responsible for attending to the low-income population in Brazil).

### Diagnosis and treatment

All participants reported that they made the diagnosis of ADHD by anamnesis, endorsed by the clinical criteria in disease classification manuals. Seventy-one (71.1%) participants used the Diagnostic and Statistical Manual of Mental Disorders-fifth edition (DSM-5) and 37 (28.9%) used the International Classification of Diseases-tenth edition (ICD-10). In addition, 122 (95.3%) reported using school reports; 114 (89.1%) used questionnaires such as the Swanson, Nolan, and Pelham-IV scale (SNAP IV); and 12 (9.4%) participants used other instruments.

Once the diagnosis was established, 123 (96.1%) participants reported referring the patient for assessment and/or treatment to a multidisciplinary team (psychologists, speech therapists, psycho-pedagogists, and occupational therapists). Among these professionals, most of the neurologists referred the patients to psychologists (n = 118, 95.9%).

A greater symptomatic improvement was perceived following combination treatment (combination of medication and intervention by a multidisciplinary team) when compared with other treatment schemes, with 124 (96.9%) of the participants reporting symptomatic improvement.

The use of medications was most frequent among patients aged 7–10 years (108 replies, 84.4%) when compared with those aged > 10 years and < 7 years, which corresponded to 15 (11.7%) and 5 (3.9%) replies, respectively. Short-acting methylphenidate was the most frequently prescribed medication (n = 109, 85.2%), followed by long-acting methylphenidate (n = 69, 53.9%) (Table 1).

**Table 1.** Medications most commonly used by pediatric neurologists for attention deficit hyperactivity disorder

Medication	Number and percentage of the participants
Short-acting methylphenidate	109 (85.2%)
Long-acting methylphenidate	69 (53.9%)
Amphetamine	28 (21.9%)
Others (non-stimulants)^*^	43 (33.6%)

* Tricyclic antidepressants, bupropion, clonidine.

Most of the participants (n = 65, 50.8%) indicated treatment for an indefinite duration with individualized management. When asked about continuous or intermittent use of medications in the last 12 months, 67 (52.3%) participants reported that they recommended pauses on weekends and/or during vacations, 27 (21.1%) recommended continuous treatment, and 34 (26.6%) recommended continuous use of medication as well as pauses on weekends and/or during vacations.

The most common side effect encountered by pediatric neurologists in clinical practice was headache, followed by hyporexia/lack of appetite, and weight loss (Table 2).

**Table 2.** Reported side effects of medications used for attention deficit hyperactivity disorder

Side effect	Number and percentage of the participants
Headache	99 (77.3%)
Weight loss	93 (72.6%)
Hyporexia/lack of appetite	93 (72.6%)
Anxiety	63 (49.2%)
Tic	52 (40.6%)
Insomnia	47 (36.7%)
Tachycardia	47 (36.7%)
Hyperexcitability	32 (25%)
Epileptic seizures	7 (5.5%)
Others^*^	14 (10.9 %)

* High blood pressure, mood changes, chest pain, psychotic symptoms, gastrointestinal tract symptoms.

When asked about the need to discontinue or change the medications due to low tolerability, 111 (87%) participants answered in affirmative. However, 67 (60%) participants adopted this change in less than 10% of their patients.

With respect to complementary tests before starting the treatment, 94 (73.4%) participants requested laboratory tests, 91 (71.1%) requested an electrocardiogram, and 54 (42.2%) requested an electroencephalogram.

### Statistical analysis between the groups according to the time they worked as specialists

Sixty-eight (53.1%) pediatric neurologists worked as specialists for ≤ 6 years, while 60 (49.9%) worked as specialists for > 6 years. Only one of the analyzed variables, namely “requesting a psycho-pedagogical evaluation” showed statistically significant difference between the groups.

## DISCUSSION

The use of virtual environment has advantages as well as disadvantages for data collection. The advantages include the possibility of covering participants from different geographic locations, anonymity of participants, minimization of the researcher’s influence, convenience of answering the instrument at the most appropriate time, ease of applying the instrument to several participants, obtaining large samples, minimizing typing errors once the data are entered into a virtual database, low cost, and possibility of mandatory filing of questions. The disadvantages include the possibility of e-mail being received by the participant as Sending and Posting Advertisement in Mass (SPAM), lack of skills of respondents, and dependence on technological resources and impersonality.^5,6,7^ We obtained work responses from participants belonging to all Brazilian regions and all the questions were answered. However, 10.9% of the e-mails were sent to non-existent email addresses and we believe that others may have reached the participants as SPAM. We achieved a response rate of 27.4%, which is consistent with that reported in the literature (12–25% for virtual questionnaires).^5^

All the interviewed specialists established the diagnosis of ADHD based on the criteria listed in classification systems such as DSM (71%) or ICD (28.9%).^1,8-10^ A similar survey conducted by Fitzgerald and McNicholas^11^ included 134 health professionals from seven European countries to evaluate topics such as attitudes, diagnosis, referral, treatment, and improvement in care. The responses showed similar distribution, with most of the participants (77%) using DSM as a diagnostic aid. These data highlight the importance of a solid base of updated diagnostic criteria and classification systems for mental disorders that are clinically eminent for diagnostic purposes. The preference for DSM suggests its greater clinical applicability with better characterization of symptoms compared to other classification systems, since it is a specific classification system for mental disorders. In contrast, although the version of ICD translated into Brazilian Portuguese covers all diseases, it does not contain the details of these diseases, as it is restricted to the classification of the diseases.^12,13^

Another aspect of these classification systems and diagnostic criteria is their impact on the variation in the prevalence of ADHD.^2^ Consistency in the diagnosis was observed in the present series and all respondents reported that they based their diagnosis on the clinical history while following the current classification systems and using questionnaires or school reports. Other Brazilian authors^14,15^ have observed similar results. Erbs^14^ conducted a survey involving professionals working in the field of mental health (13 psychiatrists and one neurologist). This survey aimed to evaluate the diagnosis of ADHD in the city of Joinville (Santa Catarina, Brazil).^14^ Peixoto and Rodrigues^15^ evaluated the diagnosis and treatment of school children with ADHD by mental health professionals (ten psychiatrists and ten neurologists who worked in the city of Vitória, Espírito Santo, Brazil).^15^ These authors evaluated the knowledge about ADHD among Brazilian health professionals and concluded that the diagnosis was mainly based on the DSM and/or ICD-10 diagnostic criteria.^14,15^

Pediatric neurologists in the present study uniformly understood the importance of a multidisciplinary team in the management of ADHD, with psychologists being the most commonly cited professionals. Similar results have been reported in the aforementioned surveys.^11,14,15^ Evidence of the efficacy of behavioral therapy supports the role of psychologists in the treatment of ADHD.^3,16-20^

Most of the participants (n = 124, 96.9%) reported a significant improvement in symptoms following combination treatment. Although initial results of the Multimodal Treatment of ADHD study emphasized the superiority of pharmacological treatment alone for symptomatic improvement, re-analyses and re-appraisals have highlighted the superiority of combination treatment for composite outcomes and for the domain of functional impairment.^21^ However, respondents in the present survey reported symptomatic improvement following pharmacological treatment alone compared to exclusive psychotherapeutic intervention, highlighting the importance of pharmacological treatment for ADHD.^21,22^

Analysis of the responses regarding indications of medication according to age group showed that the participants preferred pharmacological treatment for school-age children, which is consistent with the ADHD treatment guidelines that recommend the use of psychostimulants (first-line treatment) combined with behavioral interventions.^23,24^

Pharmacological treatment aims to normalize the prefrontal cortex activity by restoring the normal concentrations of dopamine and noradrenaline, which have been recognized to play a role in the physiopathology of ADHD. Thus, by strengthening the prefrontal cortical impulse, patients can recognize important stimuli and separate them from unnecessary ones, reducing hyperactivity and improving attention.^25-27^ Psychostimulants are the first-choice drugs for the treatment of ADHD and are widely used in children aged > 6 years, adolescents, and adults with ADHD.^1,3,9,10,23-26^

Methylphenidate has been the most frequently prescribed psychostimulant for children and adolescents since the 1990s, accounting for 77 to 87% of all prescriptions for psychostimulants.^28^ Methylphenidate was the most frequently indicated medication for the initial treatment of ADHD by the specialists in the present study. Short-acting methylphenidate was the most frequently prescribed medication, followed by long-acting methylphenidate and amphetamine. Similarly, short-acting methylphenidate was the most frequently prescribed medication by physicians in the survey conducted by Fitzgerald and McNicholas,^11^ although its dosing convenience differs from that of long-acting methylphenidate, which can be administered in a single daily dose.^1,11,29-31^ We believe that in addition to the possible benefits of short-acting medications such as a lower frequency of insomnia and weight loss, long-acting methylphenidate and amphetamines may eventually be prescribed less frequently, since they are more expensive and most of the respondents work at least part-time in the public health sector.^9,32-34^ However, this issue as well as others that may interfere with the choice of medication (such as the patient’s economic condition, presence of comorbidities at the time of ADHD diagnosis, and lack of response to psychostimulants) were not addressed in the present survey.

In contrast, 33.6% of participants reported the use of non-stimulant medications to treat ADHD. Approximately 30% of the children do not respond to or do not tolerate the initial stimulant and may benefit from medications such as tricyclic antidepressants, bupropion, clonidine, and atomoxetine (not commercialized in Brazil); which belong to drug classes other than methylphenidate or amphetamine.^35,36^ These drugs were found to be efficacious in the treatment of ADHD, although with a lower therapeutic response than stimulants. In addition to their use as substitutes for stimulants, these medications may be used as adjuvants in the treatment of ADHD or even for the treatment of comorbidities.^1,9,10,31,37^

Almost half of the respondents indicated treatment for an indefinite period with individualized management. Together with the recognition that symptoms of ADHD persist throughout adulthood, stimulant medications can be continued throughout the life in most of the children diagnosed with ADHD during elementary school.^1,30^ Discontinuation is indicated when the patient has been asymptomatic for approximately a year or when symptoms improve without the need for adjustments in medications. Development of side effects is another indication for discontinuing or reducing the dose of medications.^22,23,32^

The side effects of stimulants in children and adolescents are: uncommon, short-lived, and responsive to dose adjustments or tolerated with time of use (transient).^23^ Severe side effects (movement disorders such as tics, obsessive-compulsive thoughts, psychotic symptoms) are rare and reversed by discontinuation of the medication.^9,23^ Pediatric neurologists participating in this study reported headache as the most commonly encountered side effect in clinical practice, followed by hyporexia/lack of appetite and weight loss. Our findings were similar to those reported in a Brazilian study by Carlini et al.,^38^ which was sponsored by a pharmaceutical company for the evaluation of main side effects of methylphenidate. Altogether, 7,500 questionnaires were sent to neurologists and psychiatrists and 892 (11.9%) questionnaires were answered. It is important to emphasize that we discussed the side effects of all medications used to treat ADHD, while the survey by Carlini et al.^38^ evaluated the side effects of methylphenidate.

Possibly, the most frequently reported side effects are due to the use of short-acting methylphenidate, since it is the most frequently prescribed medication. We observed an agreement between the main side effects reported by the participants and those commonly described in clinical studies. In a randomized, double-blind, placebo-controlled trial, Greenhill et al.^39^ found that among patients using methylphenidate, the most frequent side effect was headache, followed by lack of appetite, stomach pain, and insomnia.

There are guidelines for requesting complementary tests to monitor possible side effects. Some guidelines recommend obtaining a detailed clinical history including personal or family history of cardiovascular diseases, presence of tics, and sleep disorders (insomnia) before starting pharmacological treatment for ADHD; since these conditions can be aggravated by the treatment for ADHD, especially when psychostimulants are used.^40,41^ During physical examination before starting the medication, it is important to measure weight, height, blood pressure, and heart rate to determine the exact time of occurrence of the main side effects and to carry out the correct management as previously described.^9,40,41^ Ninety-four (73.4%) participants in the present study reported requesting laboratory tests and 91 (71.1%) requested an electrocardiogram. According to Cortese et al.,^32^ there is no evidence suggesting that pharmacological treatment with psychostimulants is associated with alterations in the QT interval, sudden cardiac death, acute myocardial infarction, or cerebrovascular accidents. Some of the main guidelines for the treatment of ADHD (American Academy of Pediatrics, Canadian ADHD Practice Guidelines, National Institute for Health and Clinical Excellence Guideline) and some systematic reviews do not recommend a routine electrocardiogram. It is indicated only in case of family and/or personal history of cardiovascular diseases, a history of sudden death among first-degree relatives, risk of QT-interval alteration by the medication of choice, and changes in cardiac physical examination.^40-45^ Since the participants were not asked about the reason for requesting the exam, we believe that some requests for complementary electrocardiograms were due to a family or personal history of heart diseases.

Fifty-four (42.2%) specialists reported requesting an electroencephalogram before starting the treatment. These participants believed that psychostimulants may reduce the seizure threshold. According to Kaufmann et al.,^46^ methylphenidate, the main psychostimulant used to treat ADHD, can cause sleep deprivation and reduce the seizure threshold. However, it does not exert any effect on neurotransmitters such as gamma-aminobutyric acid, glutamate, and aspartate or on calcium and sodium, which are associated with the physiopathology of epilepsy. These findings suggest that methylphenidate does not increase the risk of epileptic seizures.^46^ Controlled clinical trials have demonstrated the safety and efficacy of methylphenidate in children with both epilepsy and ADHD.^33,47-50^ These studies have concluded that methylphenidate can be used safely in patients with ADHD without epilepsy, with controlled epileptic seizures, or with electrographic alterations in the absence of clinical seizures. In patients with uncontrolled epileptic seizures, careful clinical follow-up and electrographic monitoring are necessary during treatment.^50^

The last debatable point in the management of patients was continuity of treatment. Considering the heterogeneity of the disorder, we found different management practices among our participants regarding the indication of continuous use of psychostimulants or pausing on weekends and/or during school vacations. Pausing the medication is generally indicated when the symptoms compromise school performance due to side effects such as insomnia, lack of appetite, weight loss, and growth retardation.^1,9,22,23,32^

The two groups of specialists exhibited similar behavior regarding requests for evaluation and therapy by a multidisciplinary team. However, request for psycho-pedagogical evaluation was more frequent among pediatric neurologists who worked as specialists for ≤ 6 years, which could be attributed to concomitant learning disabilities at the time of ADHD diagnosis (a prevalence of 10 to 25%).^51,52^

### Limitations

It was not possible to accurately determine the response rate. Hence, we could not extrapolate these behaviors to all Brazilian pediatric neurologists due to methodological limitations.

## CONCLUSION

The present study evaluated aspects of ADHD considered important by researchers. These included diagnostic resources as well as questions regarding treatment such as the role of a multidisciplinary team, most suitable drugs, reasons for discontinuing treatment, most common side effects, requests for complementary tests before starting the medications, and continuity of treatment. Brazilian pediatric neurologists participating in this study complied with clinical guidelines as well as guidelines regarding pharmacological and non-pharmacological treatments, emphasizing the importance of diagnostic manuals and treatment guidelines for an eminently clinical situation and enabling uniformity in quality treatment.
